# Evaluation of Safety of Medical Trainees on Global Health Rotations

**DOI:** 10.4269/ajtmh.22-0279

**Published:** 2022-12-12

**Authors:** Noah Kojima, Jesse Ross, Christopher Tymchuk

**Affiliations:** ^1^ Department of Medicine, University of California Los Angeles, Los Angeles, California;; ^2^ Department of Medicine, Columbia University, New York, New York

## Abstract

We conducted a survey on the health and safety of medical trainees who participated in a short-term international clinical elective at a large academic training institution. We distributed an anonymous 28-question online survey via e-mail to the 142 participants available who, together, completed 185 international clinical electives. Of the 142 participants sent an anonymous survey, we received 68 responses (response rate, 48%). Of the respondents, 41 (61%) reported experiencing some form of illness. Of those, two respondents (5%) reported seeking care from a medical physician. The most commonly reported adverse health events were diarrhea (*n* = 32, 48.5%); fever (*n* = 13, 19.4%); a cough, cold, or flu-like illness (*n* = 9, 13.4%); and vomiting (*n* = 7, 13.6%). There were no reported needlestick injuries or motor vehicle accidents, and none of the reported adverse health events led to hospitalization or early termination of the elective. Four participants (5.9%) reported concerns of personal property and two (2.9%) were victims of a robbery. Two participants (2.9%) reported concerns of physical safety; however, no one reported being a victim of physical assault. Although the majority of respondents reported experiencing some form of illness, the vast majority were minor and self-limited in nature. Further studies are needed to assess problems related to mental health on international rotations and whether interventions could be used to decrease the rates of illness among participants of short-term international clinical electives.

## INTRODUCTION

Short-term international clinical electives continue to grow in popularity among medical trainees.^1^ Several studies have demonstrated interest in these electives, which have been shown to have educational benefits and can be one of the decisive factors when choosing internal medicine residencies.^2^^–^^7^

In a survey study of internal medicine and combined internal medicine–pediatrics residents that rotated for a short-term elective in Malawi, Africa, respondents reported that participation in the elective increased medical knowledge, altered career trajectory, and was influential in their selection of residency program.^7^ A systematic review of internal medicine and pediatric residents identified four positive themes emerging from international clinical electives: improved medical knowledge, physical exam, and procedural skills; improved resourcefulness and awareness of cost-effectiveness; improved cultural and interpersonal competency; and professional and career development.^5^

Despite the growing interest in international clinical electives, a formal assessment of medical trainee health and safety has not yet been conducted, to our knowledge. We sought to add to this body of literature by conducting a survey on the health and safety of medical trainees after participating in a short-term international clinical elective.

## METHODS

In this self-reported observational study, we assessed the health and safety of University of California Los Angeles (UCLA) medical trainees that participated in global health electives between 2008 and 2019.

### Setting.

The UCLA Department of Medicine’s Global Health Pathway offers short-term international clinical electives each year, with the option to rotate at partner sites in Malawi, Peru, and Thailand. All medical trainees underwent a mandatory predeparture orientation, which consists of lectures about health and safety, and a pretravel visit with a travel medicine physician. During the elective, medical trainees are supervised directly onsite by a UCLA faculty member or a local, vetted practicing physician for the majority of their elective.

### Participants.

The study population included medical students, residents, and subspecialty fellows that participated in global health electives. All participants had received a medical license or were practicing under a local physician’s license with their supervision. An archive of e-mail addresses provided to the program with medical trainee permission was used to communicate with prior participants.

Before the medical trainees went to their global health elective site, most attended a travel clinic, obtained international insurance, and underwent training in a predeparture orientation. Trainees were educated about and supplied with postexposure prophylaxis for HIV infection.^8^ In addition, trainees were given 24-hour emergency contact numbers for support from physicians at their home institution.

### Survey.

An Internet survey was developed for this study (SurveyMonkey, San Mateo, CA). The survey consisted of 28 questions that collected information regarding the international site, any experienced health and safety problems, as well as perceived health and safety problems. The survey was continued through early 2020, when international electives for trainees were put on hold by the University of California. Data were compiled on Microsoft Excel (Microsoft Corp., Redmond, WA). The survey was approved by the UCLA institutional review board (No. 15-001943-PAR-00000001).

## RESULTS

Of the 160 total participants who participated in a short-term international clinical elective between 2008 and 2019, 142 participants provided an active e-mail address and agreed to be contacted after graduation from the residency program. We distributed an anonymous, 28-question online survey via e-mail to the 142 participants available who had together completed a total of 185 international clinical electives.

Of the 142 participants sent an anonymous survey by e-mail, we received 68 responses (response rate, 48%). The majority of respondents were third-year residents who trained in internal medicine or internal medicine–pediatrics at the time of their international rotation (Table 1). Of the respondents, 67 (99%) reported providing direct patient care and 62 (91.2%) respondents had a 4-week elective. The majority of respondents rotated at a UCLA internal medicine partner site in Lilongwe, Malawi (*n* = 60, 88%); and the remainder at partner sites in Iquitos, Peru (*n* = 7, 10%); and Bangkok, Thailand (*n* = 1, 2%).

**Table 1:** Characteristics of survey respondents who attended an international elective through an internal medicine department at a large academic intuition between 2008 and 2019

Variable	*n*	%
Year of training		
Medical student	9	14.5
PGY2	3	4.8
PGY3	38	61.3
PGY4	6	9.7
Fellow	6	9.7
Specialty		
Internal medicine	49	72.1
Medicine–pediatrics	12	17.6
Infectious diseases	4	5.9
Medical student	1	1.5
Other	2	2.9
How well were you prepared for the elective?		
Not at all	0	0.0
A little	5	7.4
Moderately	11	16.2
A lot	21	30.9
Quite a lot	28	41.2
N/A	3	4.4
Did you attend predeparture orientation?		
Yes	66	97.1
No	2	2.9
Did you register for travel insurance through the university?		
Yes	58	87.9
No	4	6.1
Not aware of the option	4	6.1
Did you visit a travel medicine clinic?		
Yes	49	73.1
No	18	26.9
What vaccines did you receive prior to your elective?		
Hepatitis A	20	29.9
Hepatitis B	9	13.4
Typhoid	47	70.1
Yellow fever	24	35.8
Measles/mumps/rubella	8	11.9
Polio	8	11.9
Rabies	1	1.5
Tetanus/diphtheria	8	11.9
Japanese encephalitis	2	3.0
None of the above	8	11.9
Other; please specify	11	16.4
Was malaria prophylaxis indicated for your elective?		
Yes	67	98.5
No	1	1.5
If malaria prophylaxis was indicated for your elective, did you take malaria prophylaxis?		
Yes	67	98.5
No	0	0.0
N/A	1	1.5
During your elective, did you provide direct, hands-on patient care?		
Yes	67	98.5
No	1	1.5
During your elective, did you have any of these illnesses? Please select all that apply.		
Fever	13	19.4
Cough, cold, or flu-like illness	9	13.4
Asthma exacerbation	0	0.0
Diarrhea	32	47.8
Vomiting	7	10.4
Abscess	1	1.5
Fracture	0	0.0
Cut/laceration	4	6.0
None of the above	26	38.8
Other	1	1.5
If you had an illness during your elective, did you seek medical care from a clinician?		
Yes	2	3.0
No	37	56.1
N/A	27	40.9
If no, did you self-treat the illness?		
Yes	30	45.5
No	7	10.6
N/A	30	45.5
If you had an illness during your elective, did your illness result in your elective being shortened or you returning home sooner than planned?		
Yes	0	0.0
No	39	59.1
N/A	28	42.4
If you had an illness during your elective, did your illness require hospitalization?		
Yes	0	0.0
No	39	59.1
N/A	28	42.4
During your elective, did you have any of these exposures? Please select all that apply:		
Needlestick injury	0	0.0
Exposure to patient’s bodily fluids (e.g., exposure of patient’s blood to your eyes or oral mucosa)	4	5.9
TB exposure (e.g., examining known TB-infected patient without wearing a mask	14	20.6
None of the above	50	73.5
If you had either a needlestick injury or exposure to patient’s bodily fluids, did you take HIV postexposure prophylaxis?		
Yes	2	3.0
No	2	3.0
N/A	63	95.5
Were you diagnosed with any of the following during your elective or within 3 months of completing your elective? Please select all that apply:		
Traveler’s diarrhea	11	16.2
PPD/IGRA conversion	0	0.0
Malaria	0	0.0
Dengue	0	0.0
Zika	1	1.5
Chikungunya	0	0.0
Salmonella infection	0	0.0
None of the above	55	80.9
Other illness related to your travel, please specify	1	1.5
Did this diagnosis require hospitalization?		
Yes	0	0.0
No	22	33.3
N/A	44	66.7
During your elective, did you have any concerns for your safety or were you the victim of a crime? Please select all that apply.		
Concern for my physical safety	2	2.9
Concern for my personal property	4	5.9
Motor vehicle accident	0	0.0
Victim of robbery	2	2.9
Victim of physical assault	0	0.0
None of the above	59	86.8
Other, please specify	1	1.5

N/A = not applicable; PGY = postgraduate year; MTB = Mycobacterium tuberculosis, PPD = purified protein derivative, IGRA = Interferon-Gamma Release Assay.

Of the respondents, 41 (61%) reported experiencing some form of illness. Of those, two (5%) reported seeking care from a medical physician. The most commonly reported adverse health events were diarrhea (*n* = 32, 48.5%); fever (*n* = 13, 19.4%); a cough, cold, or flu-like illness (*n* = 9, 13.4%); and vomiting (*n* = 7, 13.6%). There was one reported Zika conversion from an elective participant at our Peru site. Of those surveyed, four respondents (5.9%) had reported exposure to bodily fluids and two of those respondents required HIV postexposure prophylaxis. HIV postexposure prophylaxis was deemed unnecessary by the supervising physician in the other reported exposures. None of our respondents reported needlestick injuries. None of the reported adverse health events led to hospitalization or early termination of the elective. There have been no reported human immunodeficiency virus infections or purified protein derivative skin test or Interferon-Gamma Release Assay conversions among the medical trainees after completing their elective.

Among those surveyed, there were four participants (5.9%) who reported concern for personal property and two (2.9%) who reported being a victim of robbery. There were two participants (2.9%) who reported concern for physical safety, but none who reported being a victim of physical assault. One participant reported harassment at an airport in transit to their international elective site. There were no reported motor vehicle accidents or physical assaults experienced by trainees during their participation in the global health electives.

## DISCUSSION

We conducted a survey on the health and safety among medical trainees who participated in a short-term international clinical elective at a large academic training institution from 2008 to 2019. Nearly everyone participated in direct hands-on patient care. Among our sample of mostly internal medicine and internal medicine–pediatric residents, we found that the majority of respondents reported experiencing some form of illness, but these were mostly mild and self-limited illnesses, with diarrhea; fever; cough, cold, or flu-like illness; and vomiting the most commonly reported medical illnesses. No participant reported a HIV, PPD, or IGRA seroconversion and there were no reported cases of malaria.

In this short-term training program, nearly everyone provided direct hands-on patient care in settings with a high clinical volume. Medical trainees in this program could have more exposure to contagious illnesses as a result of the high volume of clinical care; however, compared with other studies among travelers to Africa, respondents reported low rates of serious illnesses such as malaria.^9^ When compared with international travelers, respondents reported greater rates of gastrointestinal disease.^10^ However, because of the sampling method used in our study design, we may have captured a larger number of nonsevere cases of gastrointestinal disease.

A prior narrative review outlined risks of international clinical electives in resource-poor settings that included exposure to infectious illnesses, trauma, sexual health problems, excessive sun exposure, mental health issues, and crime.^11^ The participants of our survey fortunately did not experience any trauma or other severe medical problems. Although our respondents did not have these problems, the narrative review recommends the importance of preparation by using Web-based resources and predeparture checklists, adhering to road safety advice, taking personal safety measures, and understanding cultural awareness. Another systematic review recommended strategic site visits, comprehensive predeparture training programs, travel health assessments, and postreturn debriefing and health screening sessions to ensure travelers were healthy and safe during international electives.^12^

Our study has limitations. The survey was limited to discreet choices; therefore, we may not have assessed some areas of health and safety. Our study may have also been affected by respondent bias (as a result of the low response rate) and recall bias. Most respondents completed their global health rotation at one site, which may decrease the generalizability of our findings. We did not assess rates of comparable problems among medical trainees who were not traveling internationally, so we are unable to assess whether international travel is more dangerous than routine clinical training.

## CONCLUSION

We assessed the health and safety of medical students, residents, fellows, and attending physicians that participated in an international elective though the Department of Internal Medicine at a large academic center from 2008 to 2019. We found that although the majority of respondents reported experiencing some form of illness, the vast majority were minor and self-limited in nature, with diarrhea being the most common. Further studies are needed to assess problems related to mental health on international rotations and whether interventions could be used to decrease the rates of illness among participants of short-term international clinical electives.
